# Functional Optimization of a Novel Gluten-Free Bread Made with Tapioca Starch and Red Lentil Flour

**DOI:** 10.3390/foods15071230

**Published:** 2026-04-03

**Authors:** Federico Bianchi, Luca Agnolin, Barbara Simonato

**Affiliations:** Department of Biotechnology, University of Verona, Strada le Grazie 15, 37134 Verona, Italy; lucaagnolin@icloud.com (L.A.); barbara.simonato@univr.it (B.S.)

**Keywords:** tapioca starch, red lentil flour, mixture design, gluten-free bread, optimization study

## Abstract

Commercially produced gluten-free bread has gained popularity over the past decade. However, it often struggles to match its gluten-containing counterparts in terms of nutritional aspects, appearance, texture, and consumer acceptability. In this study, we aimed to optimize a novel gluten-free formulation based on tapioca starch and red lentil flour using a D-optimal mixture design. According to our findings, the swelling power and oil-holding capacity of the blended flour increased with the proportion of red lentil flour. The volume of bread loaves with 15% red lentil flour and 15% or 30% tapioca starch was close to that of the control bread. The addition of lentil flour tended to reduce the springiness of the experimental bread crumb, while the hardness was lower for the experimental sample with 15% lentil and 30% tapioca starch. The predicted glycemic index of the bread samples ranged from 70 to 87, and the sample containing 30% tapioca and 30% red lentil flour achieved the lowest score. Finally, the optimized gluten-free bread formulation showed lower hardness, pore density, and predicted glycemic index, and higher volume compared to the control bread sample, demonstrating that it is possible to improve gluten-free baked goods without compromises.

## 1. Introduction

The consumption of gluten is harmful to people who have celiac disease, and the only solutions consist of a gluten-free diet. However, celiac disease is just the tip of the iceberg, as gluten consumption can lead to several gluten-related disorders like dermatitis, gluten ataxia, and non-celiac gluten sensitivity [1].

Even though gluten-free (GF) bakery products are uncommon among consumers worldwide, who purchase “traditional” wheat-based bakery products, there is a growing demand for GF baked goods. In fact, the global demand for GF products has increased by 16% from 2018 to 2022 [2]. This trend can be partially explained by a rising awareness of gluten intolerance-related conditions (such as celiac disease or non-celiac sensitivity), but also by the increased consumption among people who do not suffer from any gluten-related conditions. Hassan et al. (2024) studied consumers’ perceptions of the availability, price, quality, and knowledge about gluten-free products [2]. They concluded that limited availability, higher costs, and inferior sensory attributes of gluten-free products affect consumer preferences and choices.

Therefore, developing higher-quality and more accessible GF products is decisive for improving consumer acceptance and adherence to a GF diet [2].

From a food technology perspective, the absence of gluten is a significant issue, and other components are necessary to maintain an ideal texture, volume, crumb appearance, shelf life, and overall good sensory quality. These “gluten-substitute” ingredients include hydrocolloids (e.g., gums and fibers), sourdough (lactic acid fermentation), or enzyme addition (e.g., transglutaminase) [3].

Tapioca flour and starch have gained significant attention in bread-making due to their unique properties. Indeed, they offer a neutral flavor and odor and also provide excellent thickening and binding properties [4,5]. Milde et al. (2012) investigated the use of a composite blend of tapioca starch and corn flour (80:20) [6]. They developed a spongy and high-volume bread with satisfactory sensory properties and 84% acceptability among wheat bread consumers and 100% among celiac individuals [6]. Sigüenza-Andrés et al. (2021) demonstrated that the addition of 10% to 20% of cassava products improved bread quality and volume, and they reported that newly developed Cassava starch breads were softer compared to the control breads, even after one week [7]. Additionally, Padalino et al. (2016) highlighted that tapioca flour and starch prevent bread from excessive dryness and crumbliness while acting as binders to improve cohesion and stability in gluten-free bread formulations [8].

GF bakery products, and especially bread, which are primarily based on starches, generally have poor nutritional value. For instance, GF foods have been reported to exhibit a higher glycemic index, lower protein and fiber content, and higher total and saturated fat content compared to their gluten-containing counterparts [9]. However, the use of fiber-rich flour or raw pseudocereals and legumes in GF formulations might lower the glycemic index of GF bread and improve the nutritional profile [10]. In addition, many people with celiac disease often follow an unbalanced diet, characterized by a high intake of saturated fatty acids and sugars, while also lacking important nutrients such as magnesium, iron, calcium, zinc, B12, and B9 vitamins [11].

Lentil flour can be incorporated into GF formulations to enhance the final product’s nutritional profile. Indeed, Lentils are excellent sources of protein (21–31%), dietary fiber (7–18%), as well as vitamins (B9/folate, thiamine, and niacin) and minerals (calcium, potassium, and magnesium) [12]. Lentils also contain bioactive components (polyphenols, flavonoids, and phytosterols), which are known as secondary metabolites with various functional effects [13]. In addition, legume flours affect the structure, texture, and sensory properties of the GF dough through functional effects such as water-binding capacity and solubility [14].

Considering all the above assumptions, the gluten-free bakery sector has the urgency to balance the GF bread formulations and the ingredient selection to improving from one side the technological qualities (e.g., softness, staling, volume, and crumb appearance) and on the other side the nutritional aspects (e.g., protein content, glycemic index, micronutrients, and dietary fibers) of finished products. However, formulating new bakery products is rather a complex matter since it requires combining different ingredients, often in varying proportions, and using different preparation or cooking methods. In this sense, D-optimal mixture design is a helpful optimization technique for investigating food component combinations and process variables whilst minimizing efforts, raw materials and financial costs [15].

The present study aimed to improve the textural, physiochemical, and nutritional aspects of a standard GF bread (made of cornstarch and rice flour) by substituting a mixture of lentil flour and tapioca starch. We evaluated the impact of this substitution on the novel GF loaves using a D-optimal mixture design. In addition, we evaluated the flour blends for their physicochemical properties. Finally, we optimized the formulation of a novel GF bread to achieve improved crumb hardness, pore density, volume, and the predicted glycemic index (pGI).

## 2. Materials and Methods

### 2.1. Gluten-Free Bread Ingredients and Preparation

Gluten-free bread (GF) formulations were carried out by mixing rice flour and cornstarch (MF) (265 g), apple fiber (15 g), guar gum (3 g), and dry yeast (9 g). Depending on the formulation, red lentil flour (L) and/or tapioca starch (AR) were also added in varying percentages, substituting for the flour mix (MF) made of cornstarch and rice flour (ratio 28:25). We weighed all dry ingredients in a bowl of a professional mixer (Kenwood company, Chef XL KVL4100S, Havant, UK). Then, we added 285 g of tap water at 30 °C, and the dough was kneaded at speed 1 (90 rpm) for 8 min using a flat beater. Salt (6 g) and sunflower oil (18 g) were then added, and the dough was subsequently mixed at speed 2 (120 rpm) for 8 min. Once the kneading phase was complete, we weighed approximately 200 g of dough onto a “gastronorm” tray lined with baking paper and left it to rise for 2.5 h at room temperature, covered with clingfilm. Once the rising time had elapsed, the samples were placed in a preheated professional oven in static mode at 220 °C with 100% humidity. The samples were baked for 23 min, after which the baking continued in “dry mode” for 14 min to develop the crust of the GF breads. After these 14 min, the bread samples were left to cool at room temperature on a wire rack.

### 2.2. Experimental Design

We selected a D-optimal mixture design to explore the effect of tapioca starch (AR) and red lentil flour (L) on gluten-free bread. The maximum level of MF substitution was experimentally determined and set at 60%. In comparison, the maximum level of L or AR was set at 30%, as a higher rate of substitution led to stiff dough, bread structure collapse, large holes/cracks in the crumb, and poor volume development. The experimental design is presented in Table 1, expressed in coded fractions and actual weights.

### 2.3. Model Fitting

We employed a cubic model (Equation (1)) to harness the effect of the three interdependent variables (i.e., MF, L, and AR), encompassing both linear and second-order effects.

(1) $$Y=a_{1}MF+a_{2}L+a_{3}AR+a_{12}MF\times L+a_{13}MF\times AR+a_{23}L\times AR+a_{123}MF\times L\times AR$$

where Y represents the response variables of each of the nine formulations of GF bread, and a_1_, a_2_, a_3_, a_12_, a_13_, a_23_, and a_123_ are the interaction coefficients.

The fitting of the model was conducted by R software (RStudio 2025.09.2), with a systematic procedure:

1. We appraised the initial coefficient terms and their significance.
2. Then, the model was optimized by applying a stepwise selection of linear terms based on the Akaike Information Criterion (AIC).
3. The generated models were tested for lack-of-fit (LOF) by Fisher’s F *p*-values test. The significance threshold for retaining a model was defined as *p* > 0.05.
4. We assembled the contour plots of the fitted models using Origin Pro software (OriginPro 2025b).

### 2.4. Optimization and Validation of the Optimized Formula

To generate a formulation with desired characteristics, each selected and relevant response variable was converted into a desirability function that varies from 0 to 1 (if the variable needs to be maximized) or from 1 to 0 (if the variable needs to be minimized). In the present study, the main objective was to optimize a formulation that considers important quality attributes for GF breads, including volume (maximize), hardness (minimize), pore density (minimize), and predicted glycemic index (minimize). The desirability function is then built as follows (Equation (2)):

(2) $$D=\sqrt[{n}]{d_{1}\times d_{2}\times d_{3}\times \ldots \times d_{n}}$$

where D is the geometric mean of the desirability function “d_1_, d_2_, d_3_, …, d_n_” and “n” is the number of selected response variables to optimize.

### 2.5. Flour Blends Characterization

#### 2.5.1. Water-Holding Capacity, Oil-Holding Capacity, and Water Solubility Index

We mixed three grams of each flour blend sample with 25 mL of deionized water or sunflower oil at 25 °C (for WHC or OHC tests, respectively) in a Falcon tube and shook on a wheel (UniLOOPmix, LLG, Milan, Italy) for 30 min. Tubes were then centrifuged at 1500× *g* for 10 min at 25 °C. We discarded the supernatant and weighed the pellet. WHC and OHC, expressed as grams of water or oil per gram of flour blends, were calculated as follows (Equation (3)):

(3) $$WHC\;or\;OHC\left(\frac{g}{g}\right)=\frac{p\left(g\right)-3g}{3g}$$

where p (g) is the weight of the pellet (DW) after removing the water or the oil.

The Water Solubility Index (WSI) was determined using the WHC procedure; however, supernatants were decanted onto steel plates and dried at 105 °C until a constant weight was achieved. WSI was expressed as a percentage of dried supernatants p_d_(g) compared to the initial weights (Equation (4)).

(4) $$WSI\left(\%\right)=\frac{p_{d}(g)}{3g}$$

#### 2.5.2. Water Absorption Index, Water Solubility Index of Gel, and Swelling Power

The Water Absorption Index (WAI) represents the ability of a powder to create a solid structure and retain water. The Water Solubility Index of gel (WSIG) indicates the number of compounds released in water after heating and centrifuging. The swelling power (SP) indicates the ability to retain water, typically from the starch granules, after cooking. Briefly, 1 g of the flour blend sample was mixed with 10 mL of water in a pre-weighted 15 mL Falcon tube and heated at 90 °C for 15 min. The samples were then cooled and centrifuged at 1500× *g* for 10 min. The supernatants and pellets were collected and dehydrated in pre-weighted Petri dishes at 105 °C for 24 h in a static oven. We calculated WAI, WSIG, and SP as follows (Equations (5)–(7)):

(5) $$WAI\left(\frac{g}{g}\right)=\frac{{wp}_{h}}{w_{s}}$$

(6) $$WSIg\left(\%\right)=\frac{{ws}_{d}}{w_{s}}\times 100$$

(7) $$SP\left(\frac{g}{g}\right)=\frac{{wp}_{h}}{w_{s}-{wp}_{s}}$$

where wp_h_ is the weight of the wet pellet, w_s_ is the initial weight of the sample, ws_d_ is the weight of the dry supernatant (105 °C, 24 h), and wp_s_ is the weight of the dry pellet (105 °C, 24 h).

### 2.6. Gluten-Free Bread Techno-Functional Properties

#### 2.6.1. Volume and Height

We measured the volume of gluten-free bread using an adapted “rapeseed displacement” method (AACC; Approved Methods of Analysis) [16]. A container measuring 21 × 13 × 8 cm, with a volume of 1370 cm^3^, was used. The container was initially filled with rapeseeds, the surface was leveled, and the excess rapeseeds were removed. We weighed the seeds contained in the previously calibrated container. The entire procedure was repeated three times, and the average weight was determined; thus, the density of the rapeseeds was calculated. The bread was placed in the container and filled with rapeseeds, then leveled out evenly on a flat surface. This “excess” part corresponds to the volume displaced by the bread sample, which we collected and weighed. The volume of the corresponding rapeseeds was then calculated using the following formula (Equation (8)):

(8) $$Volume\;of\;Bread\left({cm}^{3}\right)=\frac{collected\;excess\;rapeseeds}{density\;of\;rapeseeds}$$

As for the height, the loaf of bread was cut vertically in half, and the height of the central section was measured using a caliper. This procedure was repeated for each bread loaf.

#### 2.6.2. Baking Loss

Baking loss refers to the weight loss in bread during baking and subsequent cooling, primarily due to moisture loss from the product. It was calculated using the following formula (Equation (9)):

(9) $$Baking\;Loss\left(\%\right)=\frac{d-b}{b}$$

where “d” is the weight of the dough and “b” is the weight of the loaf of bread after baking and cooling down to room temperature.

#### 2.6.3. Water Activity and Moisture Content

The water activity (aw) was calculated with the Hygropalm HC2-AW instrument (Rotronic Italia, Milan, Italy). The different bread samples, including both crust and crumb, were first cut into pieces with a mixer. The measurement was carried out in triplicate at 25 °C.

We determined the moisture content according to Method 44-15A (AACC 2000), with slight modifications [16]. Briefly, 3 g of previously chopped bread were placed in special aluminum trays in an oven at 105 °C for 24 h. After the necessary time had elapsed, the trays were transferred to a closed glass bell dryer for 30 min to allow the samples to reach room temperature, thereby avoiding possible weight variations caused by moisture absorption from the air. Finally, the samples were weighed to determine their dry weight. The analysis results were expressed as a percentage of water content.

#### 2.6.4. Texture Profile Analyses

The texture profile analysis (TPA) was conducted according to Official Method AACC 74-09 [16], with slight modifications, using a TX-700 texture analyser (Lamy rheology instruments, Champagne au Mont d’Or, France) equipped with a 5 kg load cell. Each test involved two consecutive compressions with a sample deformation of 50% and a 10 s rest period between compressions. The compression probe speed was set to 1 mm/s. Each sample was de-crusted and cut into 2 cm thick slices; then, the slice was cut with a circular cutter with sharp edges, with a 2 cm diameter. For analysis, the slices were compressed using a 25 mm diameter cylindrical probe. Twelve measurements were taken for each loaf of bread sample, and values of hardness, springiness, cohesiveness, and chewiness were appraised.

#### 2.6.5. Microstructure of Gluten-Free Breads

We used the ImageJ program (version 1.54p) to analyze the alveoli in the crumb of gluten-free bread samples. The photos were taken with a Canon EOS 2000D digital camera (Canon, Amsterdam, The Netherlands), and once loaded into the program, converted to 8 bits, scaled appropriately, and the “Region of Interest” (ROI) was selected, where the analysis would be conducted (that is, an area of the bread section that did not have any crust or obvious inhomogeneities of the crumb). Subsequently, an “auto-threshold” method was applied to binarize the image, creating white objects on a black background; the image was then analyzed by the software. The parameters considered were the area of the pores, the total ROI area, and the number of pores. From some of these parameters, the pore density and pore area fraction were deducted (Equations (10) and (11)):

(10) $$Pore\;density\left(\frac{pores}{{cm}^{2}}\right)=\frac{number\;of\;pores}{ROI\;area}$$

(11) $$Area\;Fraction\left(\%\right)=\frac{\sum pore\;areas}{ROI\;area}$$

### 2.7. In Vitro Simulated Digestions of Breads

For this analysis, the crumb of gluten-free bread samples was used, which had been previously cooled and finely chopped with a mixer to simulate chewing and allow for proper enzymatic digestion. All the samples were analyzed within 16 h of production. The hydrolysis index (HI) and then the predicted glycemic index (pGI), following the protocol of Englyst et al. (1992) [17], slightly modified as reported by Rocchetti et al. (2021) [18]. This method has been validated in vitro and recognized by EFSA (European Food Safety Authority, 2011).

#### Determination of the Hydrolysis Index and the Predicted Glycemic Index

The method involves controlled enzymatic hydrolysis of starch using a mixture of pancreatic α-amylase and amyloglucosidase (AMG), followed by spectrophotometric measurement of the D-glucose released with a kit containing glucose oxidase/peroxidase (GOPOD, Megazyme, Ireland) [18,19].

The absorbance of the samples was measured at 510 nm using Tecan Infinite PRO200 (Tecan Trading AG, Männedorf, Switzerland). Soluble starch was determined using Mega-CalcM software from Megazyme, which enabled calculation of the amount of D-glucose released at different times, expressed as g/100 g, based on sample absorbance and weight. To calculate the pGI, it was necessary to construct the starch hydrolysis curve for each bread sample by graphically plotting the values of the amount of hydrolyzed D-glucose on the y-axis against 0, 20, 60, 120, and 180 min (on the x-axis). The hydrolysis curve for white bread, obtained using the same procedure, was used as a reference. This enabled the calculation of the hydrolysis index (HI), defined as the ratio of the area under the curve for each experimental bread sample to that of white bread. Knowing the HI, the value of the presumed glycemic index was calculated according to the equation by Granfeldt et al. (1992) [20]:

(12) pGI=8.198+0.862×HI

### 2.8. Statistical Analysis

Data is presented as mean and standard deviation from three independent trials. The variance analysis (ANOVA) was carried out, and the significant differences between samples were determined by applying Tukey’s multiple comparison test (Tukey, HSD). Principal component analysis (PCA) was performed considering all physiochemical attributes of bread samples and flour mixture samples (for a total of 16 parameters). Data were automatically normalized, and the PCA plot was generated by Origin Pro software (OriginPro 2025b).

## 3. Results and Discussion

**Figure 1:**
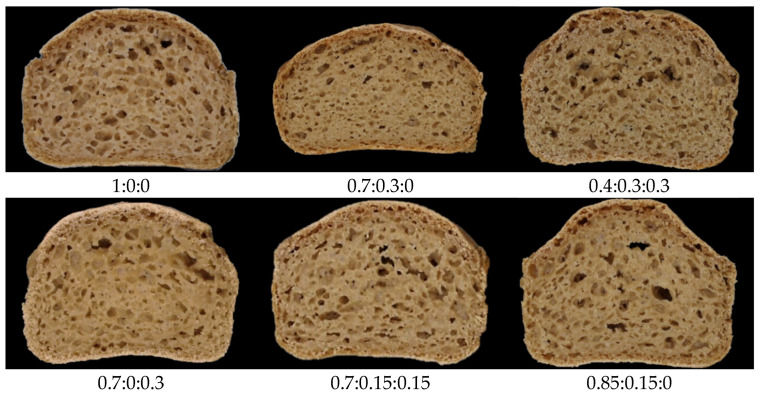

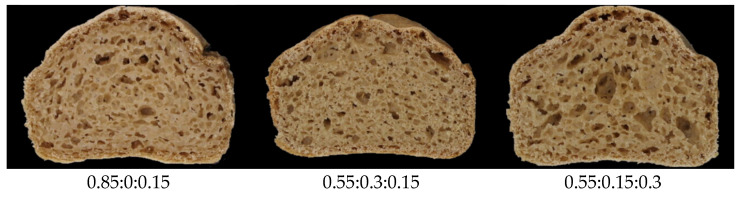
Images of experimental gluten-free bread samples. The labels reported below represent the fractions of MF, L, and AR, respectively (i.e., MF:L:AR).

In Table 2, the models’ predicted coefficients and the significances of selected variables are listed. The linear terms MFxAR and MFxARxL were not significant for any variables. We report the image of the gluten-free bread section in Figure 1. PCA (Figure S1) explained a cumulative variability of 71.4% by the first two factors (PC1 = 51.8% and PC2 = 19.6%).

### 3.1. Functional Characterization of Flour Mixtures

WHC, OHC, WSI, WAI, WSIg, and SP represent fast and easy methods for functionally characterizing flour mixtures and identifying potential correlations with finished product properties [21], and the results are reported in Table 3. In the present study, we identified strong correlations between SP and WHC, and between WAI and WSI. Additionally, we found correlations between WSIg and WSI (Figure S2). For this reason, only OHC, SP, and WSIg will be displayed, while the other figures can be observed in the Supplementary Materials section (Figure S3, Supplementary Materials).

WHC is best described as a second-order polynomial equation, while WSI is a third-order polynomial equation. Sahu et al. (2021) reported that a cubic polynomial was the best-fit equation (highest LOF) for fitting WHC and WSI of blends of maize, finger millet, and defatted soy flour [22]. We argued that differences in polynomial fitting can be related to slight variations in the adaptation of the applied methods (e.g., weight amount, centrifugal force, and temperature). In this sense, harmonization of techniques should be undertaken to enable better data interpretation and comparison across studies.

**Figure 2:**
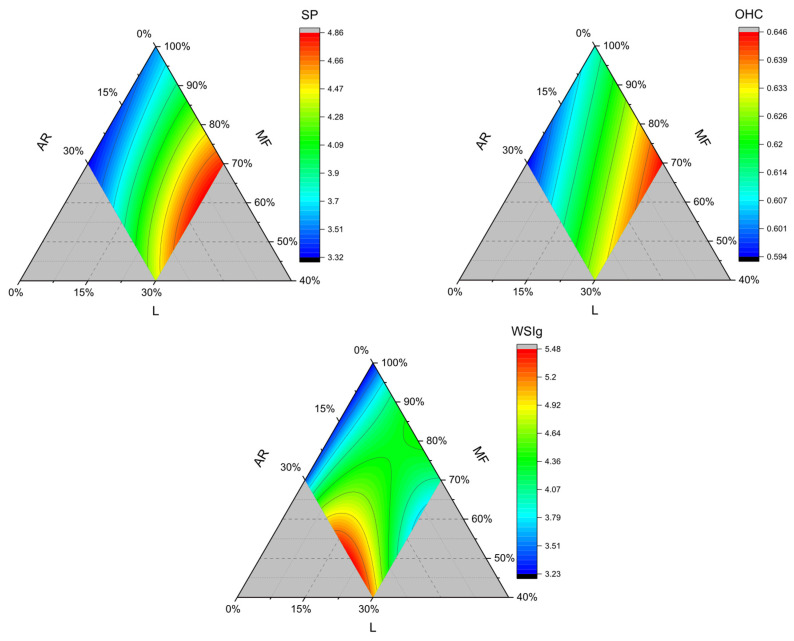
Contour plots of SP (g/g), OHC (g/g), and WSIg (%) for flour combination. AR: tapioca starch; MF: cornstarch/rice flour mixture; L: lentil flour. SP: swelling power; OHC: oil-holding capacity; and WSIg: Water Solubility Index of gels.

The swelling power of flour mixes was better described by a third-order polynomial fitting curve (Table 2). It ranged from 3.3 to 4.8 (g/g) and reached its maximum value when more than 15% lentil flour was added to the formulation, whereas its minimum value was obtained without lentil flour. The most important contribution to SP increased (Figure 2 and Table 2) is given by the positive coefficients of the term MF, AR, MFxAR, ARxL, and the cubic term ARxLx(AR–L), while negative coefficients of lentil flour and the binary combination of MFxL reduced SP of blended flours. The positive and significant contribution of the MF and AR could be related to their high starch fractions, which, upon gelatinization, promote water retention. Meanwhile, the negative contribution of lentil flour could be attributed to the protein content, which can reduce starch swelling. We agree with Lohani et al. (2026), who stated that protein and starch behave in a dualistic manner: in chickpea–cornstarch blends, starch promotes water absorption, while protein restricts swelling [23]. Similar behaviors have also been reported in lentil–corn and pea–rice systems [24,25].

At the same time, from the contour plot (Figure 2), it is evident that substituting MF with AR (while the lentil flour proportion remains constant) produces blends with lower SP. Indeed, swelling power (in the case of starch-rich ingredients) could be related to the granule’s distinctive structure as well as the amylopectin/amylose ratio [26,27]. In this sense, we can attribute the lower SP of the MF:AR blends to the higher proportion of amylopectin concentration in the mixture flour (MF). Similarly, Mauro et al. (2023) reported higher SP for rice flour and similar SP for cornstarch [27]. The same author argued that amylose content is not responsible for lowering SP or WAI. However, it is the structure of amylopectin, as well as the interaction level between the amorphous and crystalline zones [27]. On the other hand, lentil flour has less starch (Table S1) compared to MF and tapioca starch (AR). However, we may attribute L’s higher propensity to absorb water to differences in starch granules, as well as to the presence of non-starch carbohydrates that compete with AR for water during cooking.

The oil-holding capacity represents the ability of flour particles to entrap or bind oil droplets. A pure combination of the linear terms well describes the oil-binding properties of flour. According to Table 2, the most significant term is L (+0.7), followed by MF and AR. The higher OHC of L compared to AR and MF can have different explanations. For instance, Wittmüss et al. (2024) reported how oil-binding properties of commercial plant protein products (e.g., soy, sunflower) are correlated to the α-elix content of protein, their solubility, the carbohydrate content, and particle size of flours [28]. At the same time, Hopf et al. (2024) and Bianchi et al. (2025) suggested that the protein flour processing history (like methods of fractionation) and sources are influential as well [21,29]. Therefore, we can attribute lentil flour’s OHC properties to its typical carbohydrate-to-protein ratio and the size of the particles.

The Water Solubility Index of gels indicates the number of compounds released from flour blends into the water after heating and centrifugation [21], and it ranged between 4.0 and 7.5%. A third-order polynomial fit provided a better description of the WSIg of flour mixes. As reported in the contour plot (Figure 2), the higher WSIg flour mixes contain more than 15% lentil and more than 25% tapioca starch. MF, L, and AR terms contributed to increased WSIg, likely due to the presence of soluble compounds, such as soluble fibers or small and soluble oligosaccharides. The lower WSIg obtained with the flour blends at a high level of MF substitution (and low AR concentration) or a low level of L addition can be related, as previously explained, to stronger gel formation in the blended flours.

### 3.2. Techno-Functional Properties of Gluten-Free Bread Samples

#### 3.2.1. Volume and Height

**Figure 3:**
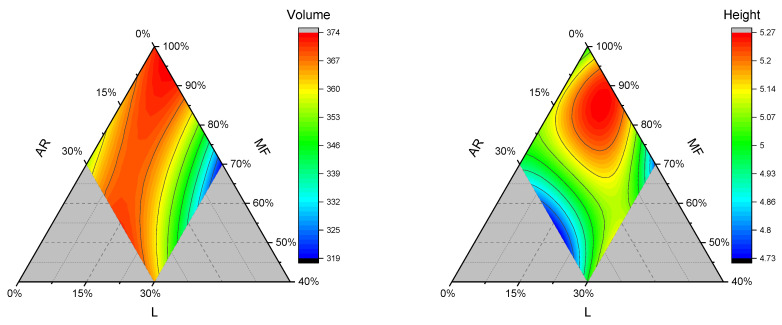
Contour plots of loaf volumes (cm^3^) and heights (cm) for gluten-free bread samples. AR: tapioca starch; MF: cornstarch/rice flour mixture; and L: lentil flour.

From Table 2, all significant terms in the volume model equation are positive, indicating that they all contribute to increasing the volume, regardless of whether they are linear, quadratic, or cubic terms. The quadratic term ARxL is significant, and the coefficient is high; thus, the addition of AR and L contributes markedly to expanding the volume of GF breads. However, the higher coefficient of the linear terms MF and AR indicates that their substitution significantly lowers gluten-free bread compared to lentil addition. Indeed, lentil flour reduces the volume of traditional bread due to interactions between gluten and lentil protein/fiber, which weaken the gluten network and thus reduce the volume [30,31]. Similar interactions may occur with the utilized hydrocolloid guar gum, which is a structuring agent with air-holding capacity for GF breads. In the present study, when L exceeds 15%, a reduction in volume is evident (Figure 1), except when tapioca starch is added. Looking carefully at the SP or WAI contour plot (Figure 3), we noticed a similar, but opposite trend in the same region. Thus, we hypothesize that the higher water absorption and swelling power of the blended flour may be responsible for the volume reduction in this constrained region. Similarly, de Pablo et al. (2025) showed that rice flour with lower water-binding capacity tended to produce GF breads with greater volumes, although the correlation was not statistically significant [32]. The same authors reported that dough consistency, as assessed by the farinograph, is a better predictor of GF bread volume [32].

At the same time, samples without lentil and with 30% of tapioca (0.7:0:0.3) showed slightly lower volume compared to control sample (1:0:0) or the 15% added lentil and no tapioca starch sample (0.85:0.15:0). Sun et al. (2015) studied the binary combination of tapioca starch and rice flour and reported that increasing proportion of tapioca rose the gel strength of the blend systems [33]. In this sense, we hypothesized that an increasing proportion of AR (in the presence or absence of a very low amount of lentil) could negatively affect the GF bread final volume due to the competing water absorption between AR and MF starch granules, which ultimately did not sustain the starch-structuring agent matrix in gluten-free bread. On the other hand, increasing the concentration of lentil food components, such as fiber and protein, may enhance bread volume [30] or interact synergistically with added hydrocolloids (i.e., guar gum), thereby improving the final structure. Moreover, lentils can contain small oligosaccharides and free sugars that can sustain yeast fermentation, improving carbon dioxide production [34].

The height of the gluten-free bread samples ranged from 4.7 cm to 5.2 cm (Table 4), with sample 0.55:0.15:0.3 being the tallest. Among the linear terms, lentil flour is the most significant, with a higher coefficient than MF and AR (12.3 vs. 5.0 and 4.1, respectively), making a fundamental contribution to increasing the height of the gluten-free bread sample. In addition, all selected terms of the cubic equation (Table 2) are positive except for MFxL, whose coefficient is negative and high. At tapioca starch concentrations above 20%, the height of GF bread decreases. However, volume is clearly unaffected in the same region, suggesting that the upper surfaces (near the crust) are flatter yet maintain a similar high volume. We observed a negative and correlated trend in the same region of the WSIg contour plot (Figure 3). We hypothesize that the increase in water-soluble compounds, such as tapioca-derived oligosaccharides or lentil-derived soluble proteins/oligopeptides, in those flour blends may partially induce an earlier crust formation (for instance, by slowing moisture migration to the crust) and therefore a flatter, less dome-shaped crust surface.

#### 3.2.2. Baking Loss, Moisture, and Water Activity

**Figure 4:**
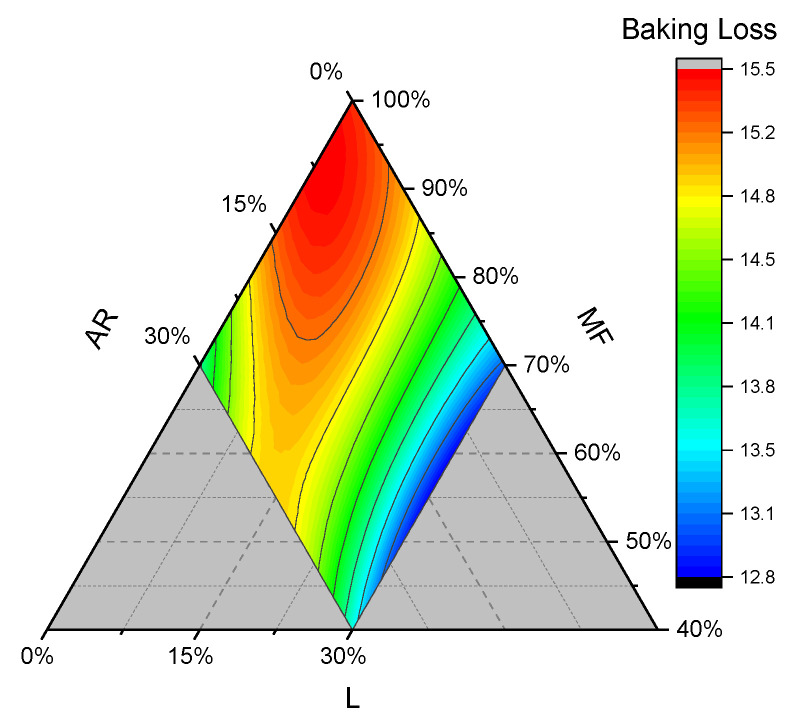
Contour plots of baking loss for gluten-free bread samples. AR: tapioca starch; MF: cornstarch/rice flour mixture; and L: lentil flour.

The average moisture content of bread samples was 44.4 ± 0.6, while the average water activity was 0.945 ± 0.006. The coefficient for the significant term in the regression equation in Table 2 indicates that MF, AR, and their combination increased BL values significantly, while L reduced them. ARxL binary combination is the highest and positive coefficient among quadratic terms, while the coefficient of ARxLx(AR—L) is the highest negative coefficient for cubic terms. In the present study, BL decreased with a higher level of lentil flour, especially when L was greater than 10%, and decreased sharply when L was closer to 30%. This effect can be attributed to a higher level of MF substitution with L, which, due to its fiber content and water-absorption capacity (SP, Figure 4; WAI, Figure S3), prevents moisture evaporation.

The mixture flour MF is the highest coefficient among linear terms: its high content is related to an increased baking loss, which can be appreciated in Figure 4, especially in the region with a low level of MF substitution, which we partially attributed to the lower swelling power of MF compared to L and AR. In addition, we noticed that the baking loss cubic equation (with a maximum around 6% of AR and 94% of MF) formed a paraboloid that visually overlaps with the one reported in the height contour plot (Figure 3). We assume, based on the previously explained hypothesis, that MF delays crust formation, thereby promoting moisture evaporation. From a calorimetric perspective, we assumed that MF starch granules (from cornstarch and rice) required a higher temperature to complete the glass transition than L and tapioca starches, thereby retarding the formation of a compact crust on the dough surface. Indeed, Joshi et al. (2013) and Saif et al. (2003) reported a peak gelatinization temperature for lentil and tapioca starch of 69 and 72, respectively, whereas Liu et al. (2006) reported cornstarch gelatinization peak around 74, while Temsiripong et al. (2005) reported a peak gelatinization temperature for rice flour around 78 [35,36,37,38]. However, it must be considered that the gelatinization peak temperature can vary depending on the initial water content, the botanical source of the plant, and starch pretreatment.

#### 3.2.3. Texture Profile Analysis

Hardness, chewiness, cohesiveness, and springiness of gluten-free bread samples are reported in Table 4. Due to a low LOF, cohesiveness modeling was unsuccessful and not displayed, while chewiness is highly correlated with hardness, and showed a very similar contour plot, and thus was reported in the Supplementary Material section (Figure S3).

**Figure 5:**
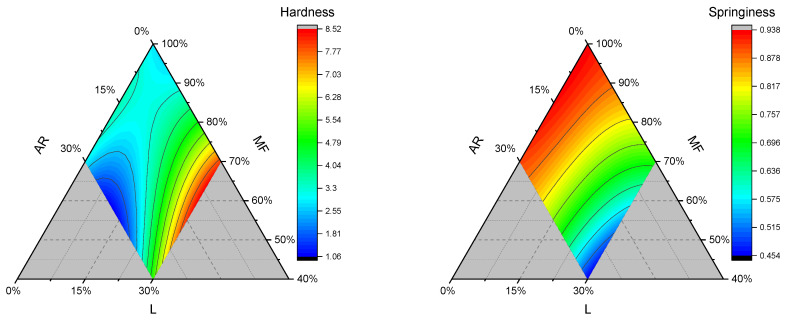
Contour plots of hardness (N) and springiness for gluten-free bread samples. AR: tapioca starch; MF: cornstarch/rice flour mixture; and L: lentil flour.

The results indicate that hardness was affected by the addition of lentil flour. Indeed, as reported in Table 2, the L linear term is the highest and is positive. Looking at Figure 5, it is evident that lentil addition increased the hardness of gluten-free crumbs, especially when the L proportion is higher than 20%, and crumb softness is dramatically reduced. This specific part of the hardness curve (L proportion > 20%) matches the BL and volume curve, suggesting that higher crumb firmness, lower volume, and less moisture evaporation can be attributed to the high level of addition of lentil flour.

MFxL and ARxL binary combinations significantly reduced the crumb hardness, and the lowest hardness was observed for the sample 0.55:015:0.3 (Figure 5). Oppositely, Portman et al. (2018) reported an increase in hardness for lentil-added wheat bread, whether they added gluten or not to the formulation [39]. However, the increased addition of AR, in the region of the curve with L > 20% and AR > 15%, decreased the hardness of the crumb of GF bread. Similarly, Kim et al. (2015) reported a lower crumb firmness with a higher proportion of tapioca starch within rice/tapioca flour blends for gluten-free bread production [40]. Therefore, we deduce that blending tapioca starch with a low concentration (less than 15%) of lentil flour is an intriguing strategy for softening gluten-free breads.

In traditional bread, springiness generally relies on the gluten network’s ability to maintain a crumb structure that bounces back after mechanical stress is applied. In the case of gluten-free bread, this capability relies on factors such as the types of starches or protein sources used and the hydrocolloids. In the present study, springiness ranged from 0.49 to 0.94 and decreased with the addition of lentil. As reported in Table 2, L is the linear term responsible for the reduction in springiness, in addition to the quadratic term ARxL and the cubic term ARxL(AR-L). Papagianni et al. (2023) studied the addition of flaxseed and sprouted/roasted lentil to gluten-free bread formulation [41]. They showed how lentil addition reduced springiness and cohesiveness, as well as increased hardness and chewiness parameters, compared to the control sample [41]. Similarly, Ammar et al. (2021) reported how whey protein concentrate reduced the springiness and cohesiveness of gluten-free bread [42]. The same authors impute these reductions to protein aggregation [42]. On the contrary, Nkurikiye et al. (2023) reported no significant springiness reduction in traditional bread added with pea, chickpea, or lentil flour, even up to 20% concentration [31].

The most significant and positive term of the fitted curve is MF (Table 2). Indeed, as the MF proportion increased, we observed a rise in springiness values (Figure 5). In addition, keeping the L term constant, the partial substitution of MF with tapioca starch (AR) reduced the springiness of gluten-free bread samples, especially at higher lentil concentrations. This effect could be ascribed to the different properties of the starch sources employed in the present study. An et al. (2022) analyzed in depth the relationship between wheat starch characteristics and noodle springiness and reported how branching rate, average width, and average length of starches affected noodle properties [43]. In addition, they found that the elasticity of compound dried noodles rose with increasing solubility and decreasing swelling power of starch [43]. Similarly, in our study, we found a negative correlation (Figure S2) between Springiness vs. WSI or WSIg. In this sense, we hypothesize that the interaction of starches with different structures and physiochemical properties can positively interact with the added guar gam and apple fibers to enhance the elasticity of the gluten-free crumbs. On the other hand, Onyango et al. (2011) reported that tapioca starch enhanced the elasticity of gluten-free bread prepared with different proportions of sorghum [44].

#### 3.2.4. Microstructure Analysis

**Figure 6:**
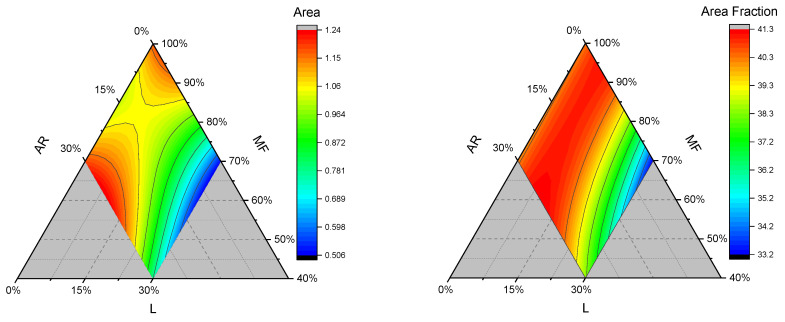
Contour plots of area of pores (mm^2^), and area fraction (%) for gluten-free bread samples. AR: tapioca starch; MF: cornstarch/rice flour mixture; and L: lentil flour.

The results of the microstructure analysis are reported in Table 5 and the contour plot in Figure 6 and Figure S2. The pore area ranged from 0.53 to 1.20 mm^2^, while the area fraction was between 33 and 40.5% for gluten-free bread samples.

The most influential linear term of the area of pores equation is L, which is negative, contributing to a decrease in pore size, while to a lesser extent, MF and AR contributed positively. Bread crumb can be described as a viscoelastic foam structure, which ideally consists of a high number of air cells in a slice, each separated by a thin wall. The interference of the structuring ability of the hydrocolloids or gums or the dilution of the starch matrix can cause a reduction in the air-retention ability of dough, thus leading to a disrupted crumb structure [45]. Lentil flour is rich in fiber and protein, which can interfere with proper crumb development, for instance, by antagonistic interaction with guar gum, or by water absorption, which increases the viscosity of the dough, thus reducing dough expansion during baking [46]. We attributed the increased volume of bread and the softness of the crumb to the pore’s density and size. As a matter of fact, in the present study, the areas of pores (and the area fractions as well) are statistically negatively correlated with hardness and positively correlated with the volume. In addition, we observed negative correlations between SP, WSI, WHC, and WSIg vs. pore area, area fraction, and density. As an explanation, we hypothesize that lower water retention or holding capacity of flour blends might promote free water that is available during baking for steam-generated air cells [47].

Regarding the area fraction, MF and AR had the highest and positive linear term coefficients, compared to L, which had a slightly negative linear term coefficient; therefore, they contributed to increasing the volume of the GF bread samples.

### 3.3. Predicted Glycemic Index

**Figure 7:**
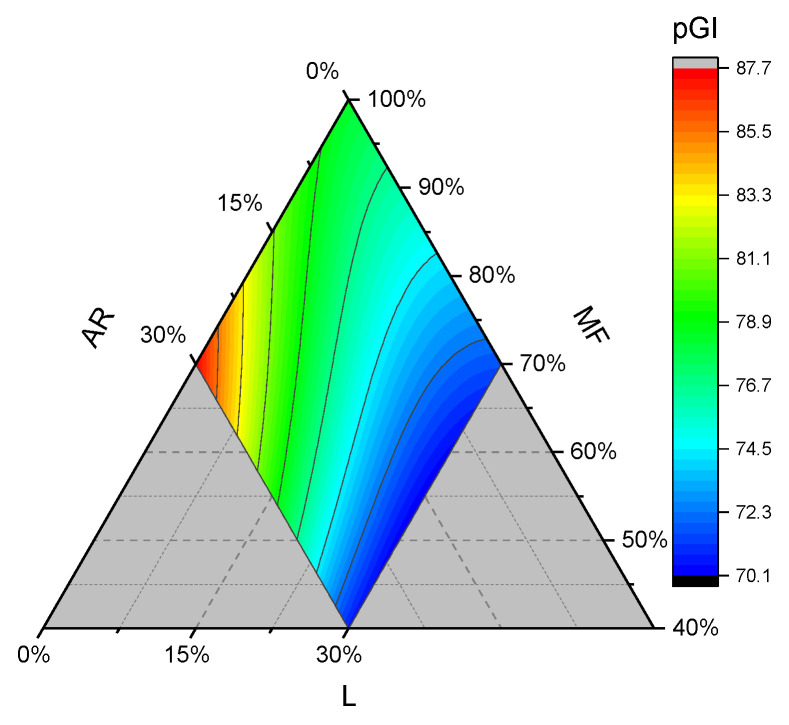
Contour plots of the predicted glycemic index for gluten-free bread samples. AR: tapioca starch; MF: cornstarch/rice flour mixture; and L: lentil flour.

The predicted glycemic indexes of bread samples are reported in Table 6, while the equation terms are n Table 2. All linear terms are positive, and AR mainly contributed to the rise in pGI, as can also be observed in the contour plot (Figure 7), while L is the linear term with the lowest contribution.

The combination of ARxL is high and negative, thus it contributed to markedly reducing starch digestion. Overall, keeping constant the tapioca starch, lentil addition was the main driving factor in pGI reduction, and we obtained the best “lower glycemic index” gluten-free bread formulation by utilizing a higher proportion of MF and L. Similarly, Zhao et al. (2023) reduced the glycemic index of gluten-free cookies with an optimized formulation with inulin and oat fiber addition [48]. The use of ingredients with higher contents of dietary fiber and protein has been shown to lower the glycemic index in gluten-free breads [49]. The main factors can be ascribed to a substitution effect with starches, as well as to the formation of a barrier between fibers/protein and starch granules, which physically hinders amylase activity. For instance, it is generally recognized that the chickpea flour’s ability to decrease the pGI is because its protein content limits the availability of starch for α-amylase by “encapsulating” it in its structures [50,51]. In addition, other mechanisms have been reported that explain the reduction in starch hydrolyzation, like the interaction of proteins with starch, therefore promoting the formation of starch-resistant assemblies, and the interaction between proteins and α-amylase [52]. In the present study, the presence of L proteins or fibers and MF starches slightly reduced the pGI compared to the AR with L blend. We deduced that starches from rice flour and cornstarch are less prone to amylase hydrolysis compared to AR. But we also hypothesize that lentil protein can form a weaker physical barrier with AR starches due to their size compared to rice and cornstarch granules, which have been reported to be slightly smaller [53].

### 3.4. Desirability Function

The quality characteristics of gluten-free bread, which determine consumer acceptance, are related to its appearance, such as loaf volume, softness, and its degree of porosity. At the same time, low glycemic diets can be associated with a reduced risk of stroke for the general population, and a beneficial effect for blood pressure, inflammatory markers, kidney function, and intestinal microbiota composition in the prediabetic and diabetic populations [54]. Therefore, not only product quality but also the consumers’ health should be considered when formulating a new gluten-free product.

**Figure 8:**
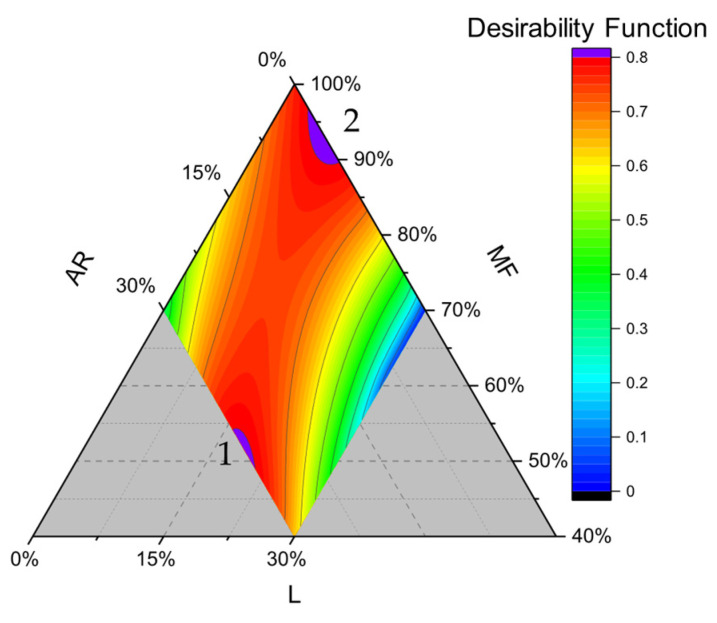
Contour plots of the desirability function considering hardness (minimized), volume (maximized), pore density (minimized), and pGI (minimized) variables. Purple sections (1 and 2) indicate a value of desirability higher than 0.8. AR: tapioca starch; MF: cornstarch/rice flour mixture; and L: lentil flour.

Thus, to determine the optimal flour composition, we optimized the GF bread formulation, considering both physical (lower hardness, higher volume, lower pore density) and nutritional aspects (lower pGI). The GF bread quality features to optimize were assigned the same relative importance, and the desirability contour plot is reported in Figure 8.

The purple section of the curve shows the selected optimum ranges of MF, L, and AR with a score higher than 0.8. Two zones stood out, the first one (1, Figure 8) ranges between 50 and 55% of MF, 15–23% of L, and a very high level of tapioca starch (AR > 28%). The second one (2, Figure 8) ranges between 90 and 97% of MF, 5–10% of L, and a very low amount of tapioca starch (AR < 5%). The maximum value is 0.82, corresponding to the formulation with 94%MF, 6% L, and 0% AR. For the validation step, we utilize this formulation, since from the consumer perspective, the label is shorter, and the pore density is lower, potentially increasing its appealability to consumers, even though the intersection 1 is characterized by a slightly lower pGI (Figure 7) and a higher concentration of protein and fibers due to a higher proportion of the lentil fraction.

In Table 7, we reported the hardness, pore density, pGI, and volume values of the prediction vs. the experimental values. The hardness, pore density, pGI, and volume of the optimized GF bread were 2.76 N, 34.6 pores per cm^2^, 75.2 predicted glycemic index value, and 369 cm^3^, respectively, which represents an improvement compared to the control sample with 100% of MF. Moreover, the quality attributes detected experimentally also confirmed the quality of the selected models.

In conclusion, we successfully developed a GF bread formulation with improved technological characteristics and a lower pGI compared to a control GF bread. However, we should note that other formulations may be worth considering, depending on the selected quality variables for the optimization steps. Future research will consider sensory evaluation and consumer acceptance analysis to confirm the commercial feasibility of the developed gluten-free bread.

## 4. Conclusions

Commercially produced gluten-free bread has gained popularity over the past decade. However, it often struggles to match its gluten-containing counterparts in terms of nutritional aspects, appearance, texture, and consumer acceptability. Mixtures of tapioca starch and lentil flour can help improve the physical qualities and nutritional profile of gluten-free bread. In this study, we aimed to optimize a novel gluten-free formulation based on tapioca starch and red lentil flour using a D-optimal mixture design. We investigated how the mixtures affect the properties of flour, as well as the textural and physicochemical characteristics, and the predicted glycemic index of the experimental bread samples. Additionally, we identified the optimal combination of red lentil and tapioca starch to achieve an experimental gluten-free bread with the lowest crumb hardness, pore density, and predicted glycemic index while maintaining the highest loaf volume. According to our findings, the swelling power and oil holding capacity of blended flours increased with the proportion of red lentil flour. We did not detect any difference between the bread samples for water activity or moisture values, with averages of 44.4% and 0.945, respectively. The volume of bread loaves with 15% red lentil flour and 15% or 30% tapioca starch was close to that of the control bread. The addition of lentil flour tended to reduce the springiness of the experimental bread crumb, while the hardness was lower for the experimental sample with 15% lentil and 30% tapioca starch. In addition, the hardness-fitting equation demonstrated that the binary terms MFxL and ARxL are responsible for the significant reduction in hardness, while the L term is the most significant for the increase in hardness. The pore density, area fractions, and area of pores were strongly correlated with hardness and loaf’s volume, suggesting that a gluten-free bread’s crumb characterized with a lower pore density, higher area fraction, and mean pore diameters is less compact and softer. The predicted glycemic index of the bread samples ranged from 70 to 87, and the sample with 30% tapioca and 30% red lentil flour achieved the lowest score. Then, we determined the optimal flour compositions to obtain a gluten-free bread with a lower hardness, pore density, and pGI, and a higher volume. The desirability function revealed two possible solutions: one with a high tapioca fraction and the other with a low tapioca fraction. We selected the formulation for the validation step with 6% lentil flour and no tapioca starch due to its potentially higher desirability score and the shortest label. The experimental values of the novel bread formulation confirmed the suitability of the fitted equation, displaying no significant differences from the predicted values. Moreover, the optimized samples showed lower hardness, pore density, pGI, and higher volume compared to the control bread sample, demonstrating that it is possible to improve the nutritional profile of gluten-free baked goods without compromising quality.

## Figures and Tables

**Table 1:** Coded fraction of each formulation and its actual weights. On the right, the design plot is reported.

	Fraction (%)			Actual Weight (g)			
GF Bread Sample	MF	L	AR	MF	L	AR	
1	100	0	0	265	0	0	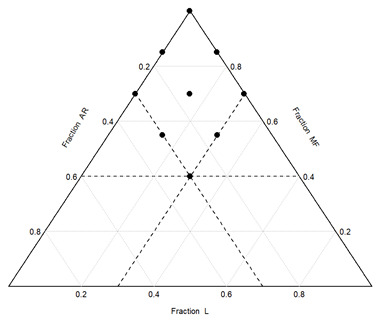
2	70	30	0	185	80	0	
3	40	30	30	105	80	80	
4	70	0	30	185	0	80	
5	70	15	15	185	40	40	
6	85	15	0	225	40	0	
7	85	0	15	225	0	40	
8	55	30	15	145	80	40	
9	55	15	30	145	40	80	

MF: cornstarch/rice flour mixture; L: lentil flour; AR: tapioca starch.

**Table 2:** Coefficient of the equation’s terms, their significances, and lack-of-fit (LOF) values of the best equation after the stepwise selection.

Dependent Variables	Coefficients of the Polynomial Terms and Their Significance								LOF (*p*-Value)
	MF	L	AR	MFxL	ARxL	MFxLx (MF-L)	MFxARx (MF-AR)	ARxLx (AR–L)	
WHC	0.9 ***	1.2 **	0.8 ***	1.4 *	-	-	-	-	0.68
OHC	0.6 ***	0.7 ***	0.6 ***	-	-	-	-	-	0.54
WSI	4.1 ***	1.7 ***	3.3 **	-	43.7 ***	22.9 ***	-	−145 ***	0.43
WAI	3.4 ***	−1.8 ***	2.7 ***	16.7 *	7.2 °°	−9.1 *	-	26.8 **	0.09
WSIg	3.2 ***	1.6 ***	3.8 ***	-	22.5 ***	15.5 °°	-	−95.1 *	0.64
SP	3.6 ***	−1.9 ***	2.8 ***	17.2 °°	8.3 *	−8.8 **	-	23.5 **	0.07
Hardness	2.8 ***	71.4 ***	−0.3	−73.3 ***	−95.6 ***	-	13.3 **	175.4 ***	0.09
Chewiness	1.2 ***	19.4 ***	−0.1	−20.6 ***	−35.6 ***	-	4.2 **	42.6 ***	0.68
Springiness	0.9 ***	−0.6 **	0.8 ***	1.2 ***	−1.1 ***	-	0.3 °	−7.5 ***	0.56
Baking loss	15.4 ***	−7.5 °°	6.1 **	21.7 *	56.6 ***	-	14 *	−130.8 **	0.60
Volume	370.5 ***	84 *	319.4 ***	-	994.8 ***	405.1 **	-	-	0.56
Height	5.0 ***	12.3 ***	4.1 ***	−17.6 ***	-	15.3 ***	2.8 *	36.5 ***	0.39
Pore Area	1.2 ***	−7 **	2.1 ***	8.6 **	8.7 ***	-	−3.3 ***	-	0.29
Density	35.5 ***	218.8 ***	−0.7	-	−300.4 ***	−261 **	113.4 ***	-	0.056
Area Fraction	40.3 ***	−1.8	39.8 ***	-	112.1 ***	66.3 ***	-	-	0.68
pGI	78.4 ***	55.9 ***	120 ***	-	−143.4 ***	-	−38.8 °	-	0.71

Level of significance: “***” *p* < 0.001; “**” *p* < 0.01; “*” *p* < 0.05; “°” *p* < 0.1; “°°” *p* > 0.1; “-“ non selected term during stepwise selection. MF: cornstarch/rice flour mixture; L: lentil flour; AR: tapioca starch; LOF: lack-of-fit; WHC: water-holding capacity; OHC: oil-holding capacity; WSI: Water Solubility Index; WAI: water absorption capacity; WSIg: Water Solubility Index of gels; SP: swelling power; and pGI: predicted glycemic index.

**Table 3:** Mean values and standard deviations of water-holding capacity (WHC), oil-holding capacity (OHC), Water Solubility Index (WSI), water absorption capacity (WAI), Water Solubility Index of gels (WSIg), and swelling power of the tested flour combinations.

MF	L	AR	WHC (g/g)	OHC (g/g)	WSI (%)	WAI (g/g)	WSIg (%)	SP (g/g)
1	0	0	0.9 ± 0.0 ^def^	0.61 ± 0.02 ^a^	4.1 ± 0.3 ^d^	3.4 ± 0.1 ^e^	3.2 ± 0.1 ^e^	3.5 ± 0.1 ^e^
0.4	0.3	0.3	1.1 ± 0.1 ^bc^	0.65 ± 0.08 ^a^	7.5 ± 0.6 ^a^	4.1 ± 0.2 ^bc^	5.1 ± 0.1 ^a^	4.4 ± 0.2 ^bc^
0.7	0.3	0	1.3 ± 0.1 ^a^	0.64 ± 0.02 ^a^	5.3 ± 0.1 ^c^	4.6 ± 0.0 ^a^	4 ± 0.2 ^bcde^	4.8 ± 0.0 ^a^
0.7	0	0.3	0.9 ± 0.0 ^f^	0.58 ± 0.06 ^a^	4.0 ± 0.3 ^d^	3.2 ± 0.1 ^e^	3.3 ± 0.2 ^de^	3.3 ± 0.1 ^e^
0.7	0.15	0.15	1.1 ± 0.1 ^cd^	0.58 ± 0.03 ^a^	5.6 ± 0.3 ^c^	3.8 ± 0.0 ^d^	4.4 ± 0.3 ^abc^	4.0 ± 0.0 ^d^
0.85	0.15	0	1.1 ± 0.1 ^abc^	0.65 ± 0.08 ^a^	6.0 ± 0.9 ^bc^	4.0 ± 0.0 ^cd^	4.4 ± 0.5 ^ab^	4.2 ± 0.1 ^cd^
0.85	0	0.15	0.9 ± 0.0 ^ef^	0.63 ± 0.02 ^a^	4.0 ± 0.2 ^d^	3.3 ± 0.0 ^e^	3.5 ± 0.6 ^cde^	3.5 ± 0.0 ^e^
0.55	0.3	0.15	1.3 ± 0.0 ^ab^	0.62 ± 0.01 ^a^	7.2 ± 0.2 ^ab^	4.3 ± 0.1 ^b^	5.2 ± 0.1 ^a^	4.5 ± 0.2 ^ab^
0.55	0.15	0.3	1.1 ± 0.1 ^cde^	0.62 ± 0.01 ^a^	5.3 ± 0.1 ^c^	4.0 ± 0.1 ^cd^	4.1 ± 0.1 ^bcd^	4.2 ± 0.1 ^cd^

Values with different superscripts within the same column are significantly different for *p* < 0.05. MF: cornstarch/rice flour mixture; L: lentil flour; and AR: tapioca starch.

**Table 4:** Mean values and standard deviations of loaf volume, height, baking loss, hardness, springiness, cohesiveness, and chewiness of the experimental bread samples.

MF	L	AR	Volume (cm^3^)	Height (cm)	Baking Loss (%)	Hardness (N)	Springiness	Cohesiveness	Chewiness (N)
1	0	0	369 ± 14 ^a^	5.0 ± 0 ^b^	15.4 ± 0.5 ^a^	2.93 ± 0.35 ^d^	0.94 ± 0.01 ^a^	0.57 ± 0.05 ^a^	1.58 ± 0.24 ^b^
0.4	0.3	0.3	319 ± 9 ^c^	4.8 ± 0 ^cd^	13.1 ± 0.3 ^c^	8.09 ± 1.28 ^a^	0.79 ± 0.02 ^e^	0.41 ± 0.02 ^c^	2.59 ± 0.23 ^a^
0.7	0.3	0	366 ± 15 ^ab^	5.0 ± 0.1 ^b^	13.6 ± 0.3 ^bc^	5.25 ± 0.86 ^b^	0.49 ± 0.03 ^g^	0.3 ± 0.01 ^d^	0.79 ± 0.13 ^d^
0.7	0	0.3	356 ± 8 ^ab^	4.9 ± 0.1 ^bc^	13.8 ± 0.6 ^bc^	3.05 ± 0.35 ^cd^	0.9 ± 0.01 ^bc^	0.52 ± 0.02 ^b^	1.43 ± 0.17 ^bc^
0.7	0.15	0.15	357 ± 2 ^ab^	5.1 ± 0.1 ^ab^	15.1 ± 0.3 ^a^	3.88 ± 0.55 ^c^	0.82 ± 0.02 ^d^	0.4 ± 0.02 ^c^	1.28 ± 0.17 ^c^
0.85	0.15	0	369 ± 10 ^a^	5.2 ± 0.1 ^a^	14.6 ± 0.3 ^ab^	3.56 ± 0.86 ^cd^	0.89 ± 0.03 ^c^	0.51 ± 0.03 ^b^	1.61 ± 0.3 ^b^
0.85	0	0.15	364 ± 10 ^ab^	5.1 ± 0.1 ^ab^	15.1 ± 0.3 ^a^	3.35 ± 0.53 ^cd^	0.93 ± 0.01 ^ab^	0.55 ± 0.03 ^ab^	1.7 ± 0.24 ^b^
0.55	0.3	0.15	337 ± 9 ^bc^	4.6 ± 0.1 ^d^	14.4 ± 0 ^ab^	5.67 ± 0.43 ^b^	0.7 ± 0.02 ^f^	0.38 ± 0.02 ^c^	1.52 ± 0.12 ^bc^
0.55	0.15	0.3	372 ± 17 ^a^	5.3 ± 0.1 ^a^	13 ± 0.6 ^c^	1.83 ± 0.54 ^cd^	0.69 ± 0.05 ^f^	0.39 ± 0.02 ^c^	0.91 ± 0.15 ^d^

Values with different superscipts within the same column are significantly different for *p* < 0.05. MF: cornstarch/rice flour mixture; L: lentil flour; and AR: tapioca starch.

**Table 5:** Mean values and standard deviations of the area of pores, area fraction, and the pore density of the experimental bread samples.

MF	L	AR	Area of Pores (mm^2^)	Area Fraction (%)	Pore Density (Number/cm^2^)
1	0	0	1.17 ± 0.12 ^a^	40.4 ± 1.1 ^a^	35.2 ± 3.5 ^c^
0.4	0.3	0.3	0.53 ± 0.06 ^c^	33.1 ± 3.3 ^c^	67 ± 4.6 ^a^
0.7	0.3	0	0.84 ± 0.22 ^b^	38.8 ± 2.5 ^ab^	47.3 ± 8.5 ^b^
0.7	0	0.3	1.20 ± 0.14 ^a^	40.5 ± 0.9 ^a^	34.3 ± 3.6 ^c^
0.7	0.15	0.15	1.06 ± 0.23 ^ab^	40.4 ± 1.3 ^a^	39.4 ± 6.3 ^bc^
0.85	0.15	0	1.02 ± 0.19 ^ab^	40 ± 2.5 ^a^	41.3 ± 6.9 ^bc^
0.85	0	0.15	0.99 ± 0.12 ^ab^	39.7 ± 0.6 ^a^	40.4 ± 4.3 ^bc^
0.55	0.3	0.15	0.53 ± 0.05 ^c^	35.4 ± 0.7 ^bc^	67.2 ± 4.9 ^a^
0.55	0.15	0.3	1.14 ± 0.15 ^ab^	40.5 ± 1.1 ^a^	35.8 ± 3.6 ^c^

Values with different superscripts within the same column are significantly different for *p* < 0.05. MF: cornstarch/rice flour mixture; L: lentil flour; and AR: tapioca starch.

**Table 6:** Mean values and standard deviations of the predicted glycemic index (pGI) values.

MF	L	AR	pGI
1	0	0	78.7 ± 3.5 ^bc^
0.4	0.3	0.3	70.5 ± 3.8 ^d^
0.7	0.3	0	71.1 ± 5.2 ^d^
0.7	0	0.3	88.1 ± 5.0 ^a^
0.7	0.15	0.15	76.6 ± 4.6 ^bc^
0.85	0.15	0	75.1 ± 5.5 ^cd^
0.85	0	0.15	80.2 ± 3.1 ^b^
0.55	0.3	0.15	70.7 ± 4.3 ^d^
0.55	0.15	0.3	79.1 ± 4.6 ^bc^

Values with different superscripts within the same column are significantly different for *p* < 0.05. MF: cornstarch/rice flour mixture; L: lentil flour; and AR: tapioca starch.

**Table 7:** Experimental vs predicted mean values of the selected gluten-free bread characteristics. Control bread values are reported for quality comparison.

Variables	Prediction	Experimental (94% MF, 6% L)	*p*-Values *	Control Bread (100% MF)
Hardness (N)	2.80	2.76 ± 0.5	0.23	2.9
Pore density (pores/cm^2^)	33.5	34.3 ± 1.8	0.52	35.2
pGI	77.1	75.2 ± 3.2	0.43	78.7
Volume (cm^3^)	373	379 ± 5	0.15	369

* Appraised by a *t*-test between three independent replicas vs the predicted values of the first column (single *t*-test, α = 0.05, OriginPro 2025b).

## Data Availability

The original contributions presented in this study are included in the article/Supplementary Material. Further inquiries can be directed to the corresponding author.

## Supplementary Materials

The following supporting information can be downloaded at: https://www.mdpi.com/article/10.3390/foods15071230/s1. Figure S1: PCA biplot with loadings and scores; Figure S2: Correlation matrix of functional properties of flour blends, techno-functional characteristics of GF breads, and predicted glycemic index (pGI); Figure S3: Contour plots of WHC (g water/g flour); WSI (%); WAI (%), chewiness (N), and pore density (number of pores/cm^2^); Table S1: Nutritional components of flours utilized to bake experimental gluten-free bread samples (according to packaging labels), and the nutritional content of MF (based on the proportion of rice flour and corn starch).
