# Supplementary material for: Functional Optimization of a Novel Gluten-Free Bread Made with Tapioca Starch and Red Lentil Flour
Source: Foods. 2026 Apr 3;15(7):1230. doi: 10.3390/foods15071230 (PMC13072981; doi:10.3390/foods15071230)
Supplement: Supplementary file 1 [file foods-15-01230-s001.zip › foods-4210731-supplementary.pdf]

Table S1. Nutritional components of flours utilized to bake experimental gluten-free bread samples (according to packaging labels), and the nutritional content of MF (based on the proportion of rice flour and corn starch).

Flour	Carbohydrates (g/100g dw)	Fibers (g/100g dw)	Proteins (g/100g dw)	Fats (g/100g dw)
Lentil flour	41.7	19.4	27.6	1.8
Rice flour	80.0	1.6	8.0	1.3
Corn starch	86.0	1.0	0.5	0.5
Tapioca starch	85.0	1.0	0.5	0.5
Mixture Flour (MF)	83.2	1.3	4.0	0.9

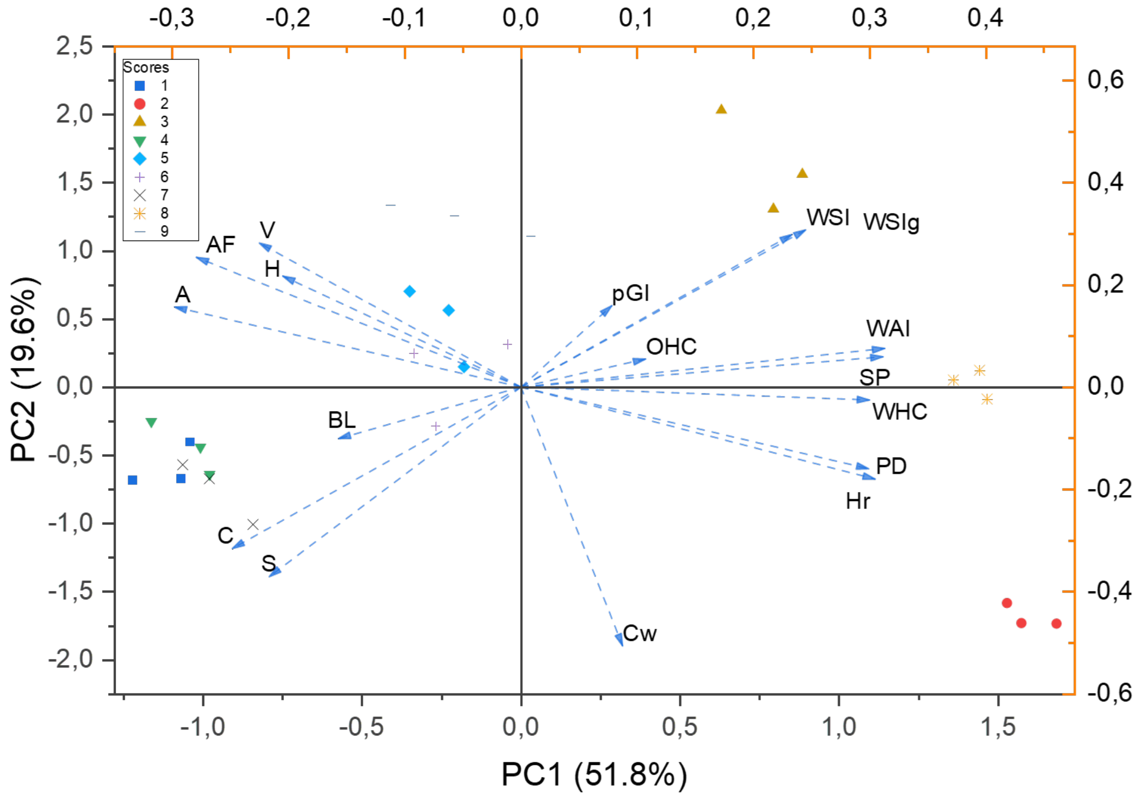

Figure S1. PCA biplot with loadings and scores. The top x-axis and right y-axis (light red) represent loading values for PC1 and PC2, respectively. Bottom x-axis and left y-axis (black) represent scores for PC1 and PC2, respectively. Samples are labeled from 1 to 9 according to the following table.

	Fraction (%)		
GF bread sample	MF	L	AR
1	100	0	0
2	70	30	0
3	40	30	30
4	70	0	30
5	70	15	15
6	85	15	0
7	85	0	15
8	55	30	15
9	55	15	30

MF: cornstarch/rice flour mixture; L: lentil flour; and AR: tapioca starch.

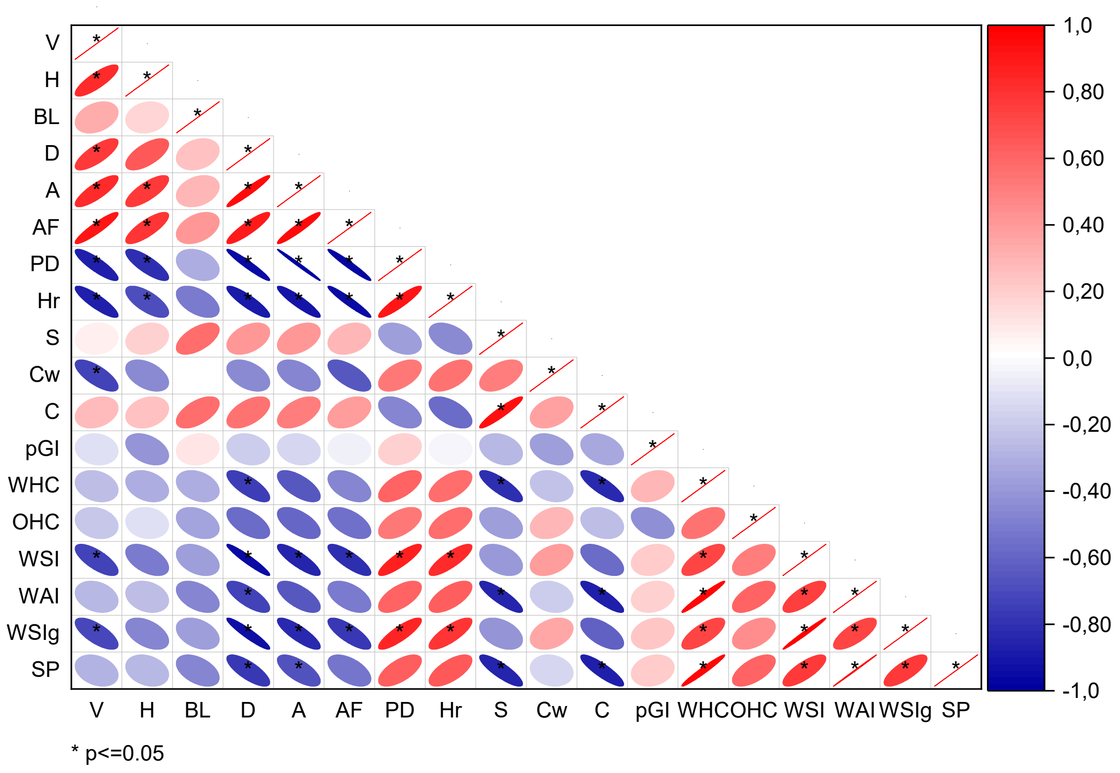

Figure S2. Correlation matrix of functional properties of flour blends, techno-functional characteristics of GF breads, and predicted glycemic index (pGI). * = significant for $p < 0.05$.

V: volume, H: height, BL: backing loss, Hr: hardness, S: springiness, C: cohesiveness, Cw: chewiness, D: diameter, A: area of pores, AF: area fraction, PD: pore density, C: crust, CB: crumb, WHC: water holding capacity, OHC: oil binding capacity, WSI: water solubility index, WAI, water absorption index, WSIg: water solubility index of gels, SP: swelling power.

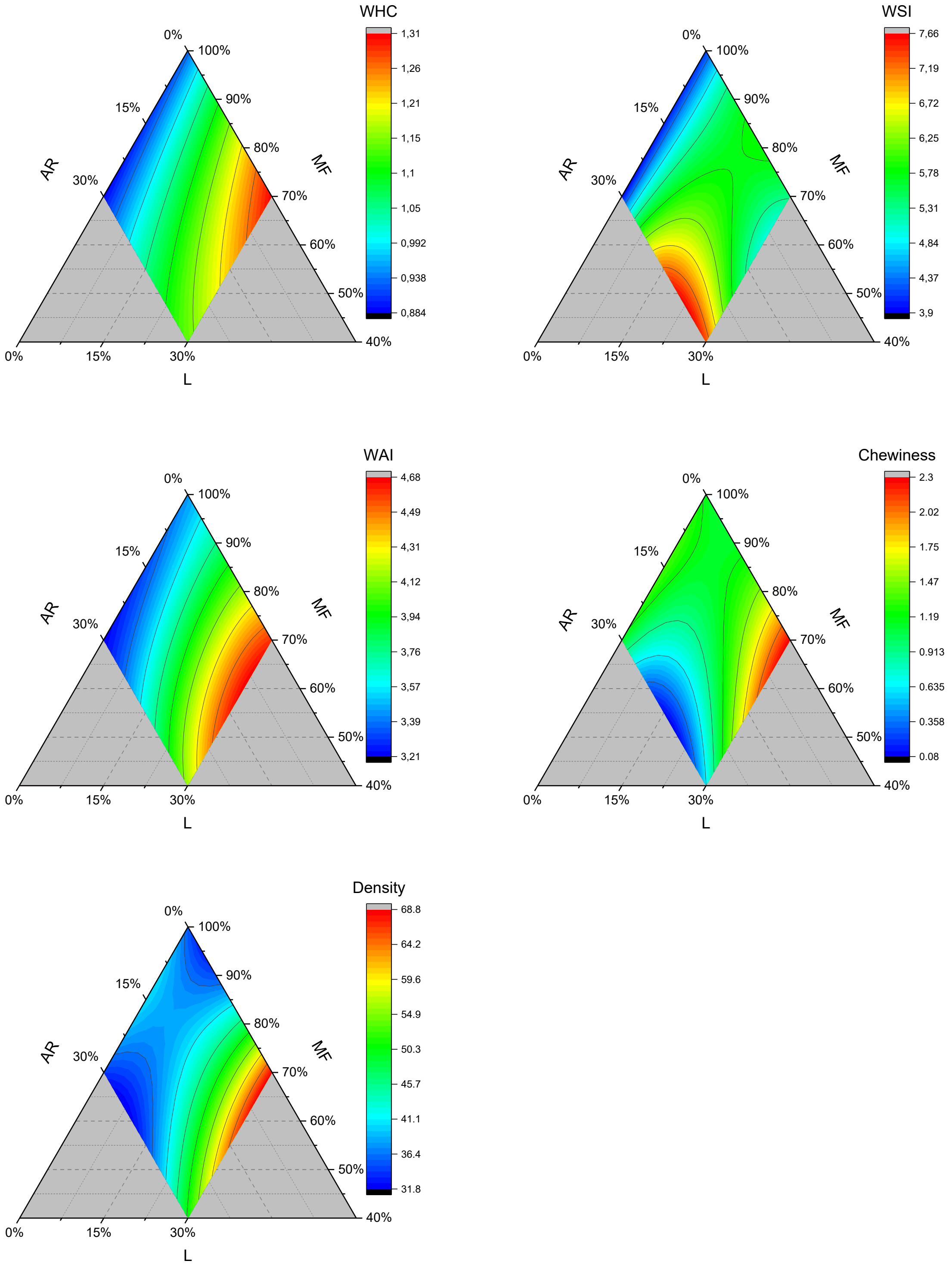

Figure S3. Contour plots of WHC (g water/ g flour); WSI (%); WAI (%), chewiness (N), and pore density (number of pores/cm^2).

AR: tapioca starch; MF: cornstarch/rice flour mixture; L: lentil flour. WHC: water holding capacity, WSI: water solubility index.
